# The distribution of sexually-transmitted Human Papillomaviruses in HIV positive and negative patients in Zambia, Africa

**DOI:** 10.1186/1471-2334-7-77

**Published:** 2007-07-16

**Authors:** Christopher Ng'andwe, John J Lowe, Paula J Richards, Lara Hause, Charles Wood, Peter C Angeletti

**Affiliations:** 1 Nebraska Center for Virology, School of Biological Sciences, University of Nebraska-Lincoln, Lincoln, Nebraska, USA; 2 University of Zambia School of Medicine, and University Teaching Hospital, Lusaka, Zambia

## Abstract

**Background:**

Human Papillomaviruses (HPV) are double-stranded DNA viruses, considered to be the primary etiological agents in cervical intraepithelial neoplasias and cancers. Approximately 15–20 of the 40 mucosal HPVs confer a high-risk of progression of lesions to invasive cancer. In this study, we investigated the prevalence of sexually transmitted HPVs in Human Immunodeficiency Virus (HIV) positive and negative patients in Zambia, Africa. The rate of high-risk HPV genotypes worldwide varies within each country. Thus, we sought to investigate the rates of HPV infection in sub-Saharan Africa and the potential role of HIV in affecting the HPV genotype distribution.

**Methods:**

This retrospective cross-sectional study reports findings on the association and effects of HIV on HPV infections in an existing cohort of patients at University Teaching Hospital (UTH) Lusaka, Zambia. The objective of this study was to assess HPV prevalence, genotype distribution and to identify co-factors that influence HPV infection. Polymerase chain reaction (PCR) with two standard consensus primer sets (CpI/II and GP5+/6+) was used to test for the presence of HPV DNA. Primers specific for β-actin were used to monitor DNA quality. Vaginal lavage samples, collected between 1998-1999 from a total of 70 women, were part of a larger cohort that was also analyzed for HIV and human herpesvirus infection. Seventy of the samples yielded usable DNA. HIV status was determined by two rapid assays, Capillus and Determine. The incidence of HIV and HPV infections and HPV genotype distributions were calculated and statistical significance was determined by Chi-Squared test.

**Results:**

We determined that most common HPV genotypes detected among these Zambian patients were types 16 and 18 (21.6% each), which is approximately three-fold greater than the rates for HPV16, and ten-fold greater than the rates for HPV18 in the United States. The worldwide prevalence of HPV16 is approximately 14% and HPV18 is 5%. The overall ratio of high-risk (HR) to low-risk (LR) HPVs in the patient cohort was 69% and 31% respectively; essentially identical to that for the HR and LR distributions worldwide. However, we discovered that HIV positive patients were two-times as likely to have an HR HPV as HIV negative individuals, while the distribution of LR HPVs was unaffected by HIV status. Interestingly, we observed a nine-fold increase in HPV18 infection frequency in HIV positive versus HIV negative individuals.

**Conclusion:**

The rate of oncogenic HPVs (type 16 and 18) in Zambia was much higher than in the U.S., potentially providing an explanation for the high-rates of cervical cancer in Zambia. Surprisingly, we discovered a strong association between positive HIV status and the prevalence of HR HPVs, and specifically HPV18.

## Background

Human papillomavirus (HPV) is the primary etiological agent causing 95% of cervical cancers. Over 200 HPV types have been recognized and approximately 40 have been shown to infect the genital tract [1,2]. Even though genital HPV infection is one of the most common sexually transmitted infections, only about 10% of people in the U.S. have active HPV infections, with 4% having cytological abnormalities and 1% showing evidence of genital warts [3]. Epidemiological evidence gathered over the last decade has designated 15–20 of the 40 mucoso-tropic HPV types (HPV16, 18, 45, 31, 33, 58, 52, 35, 59, 56, 6, 51, 68, 39, 82, 73, and 70) as associated with a high risk of progression to cervical cancer [1,2,4,5]. In addition, there is recent speculation that HPV26, 53, and 66 should also be considered high-risk strains [6-10], however, there is still some disagreement about these designations. The frequency of individual high-risk HPV types worldwide has been shown to vary in respect to major global regions such as Asia, Europe, North America, South America, and Sub-Saharan Africa [4,5,11]. Despite frequency variation, HPV16 infection has been shown to be exceedingly more prevalent than any other high-risk HPV type in these global regions. An exception to this trend has been observed in Human Immunodeficiency Virus (HIV) positive populations where HPV16 has shown to be frequent, but not as predominating as seen in most HIV negative populations [12-16]. Recent studies on the correlation between HIV and HPV infections indicate higher frequencies of high-risk HPV types in HIV positive individuals as opposed to the usual genotypic frequencies observed in HIV negative populations.

Impaired cell-mediated immunity could be a likely explanation for the advancement of HR HPVs in HIV positive individuals. Several studies have shown a strong and consistent association between human immunodeficiency virus (HIV) and HPV co-infection and the development of CIN and genital cancer [17-20]. There is evidence to show that HIV positive women have a significantly higher rate of CIN than their counterparts and are more likely to progress to invasive carcinoma than HIV negative women [21-23]. A recent study in Brazil has shown that a very high proportion of HIV infected women are infected with HPV and they often carry multiple HPV genotypes (15).

## Methods

### Participants and ethical precautions

This study reports findings from a cross-sectional analysis of data from a cohort study on the association and effects of HIV on HPV infections. All human subjects protocols were approved by safety committees at the University of Zambia and UNL in accordance with the Helsinki Declaration. Participation by patients was entirely voluntary and written patient consent was required for inclusion in the study. The objective of this study was to assess effects of HIV status on HPV prevalence, distribution of HPV genotypes and assess associated risk factors involved with HPV infection.

Zambia is part of an ongoing study site to follow HIV and secondary viral infections in women of child-bearing age at the UTH, the largest tertiary care institution in the country and the main referral center for Lusaka, the capital of the country. Between September 1998 and October 1999, female patients were approached for enrollment in the study. Those who were clinically diagnosed with KS, AIDS, TB, malaria, cancer or had any other adverse health conditions, were not eligible to participate in the study. In addition, residence in the metropolitan area of Lusaka was required. Disease histories as well as physical examinations were carried out to rule out any clinical symptoms or visible signs for these conditions. All of the participants had normal pap smears. A total of 70 vaginal lavage samples obtained from these women were analyzed,

### Demographic data collection

All study participants who signed informed consent and were evaluated by study clinicians. A set of pre-tested, standardized questionnaires was used to gather data [24]. Of 80 questionnaires 10 were not completed in full and were thus excluded from any socio-demographic analysis, leaving complete data for 70 patients. All personal identifiers were removed from samples to ensure patient confidentiality. With patients' permission, medical history was retrospectively retrieved from hospital medical records.

A total of 154 variables were identified and assessed in this study. Data were collected regarding seven areas of interest that included: **(i) **socio-demographics (age, education, household income, marital status, occupation, religion, and tribal identity); **(ii) **current medical standing (weight, diagnosis of tuberculosis, and diagnosis of specific ulcerative and non-ulcerative STDs); **(iii) **medical history (history of blood transfusion, hypertension, drug abuse, and use of antibiotics in past 12 months); **(iv) **reproductive and obstetric history (number and outcomes of pregnancies, use of family planning and birth control methods including condoms); **(v) **history of sexually transmitted disease (histories of STDs, genital ulceration, vaginal discharge, and cancer including cervical dysplasia); **(vi) **sexual behavior history (age at first sexual encounter, steady partner in past 3 years, new partners in past 3 years, sex with partner with penile lesion, sex under influence of alcohol, anal intercourse, being raped, practice of dry sex, and use of herbs vaginally; and **(vii) **laboratory test results (pap smears, results of serological testing for HHV-8, HIV-1, and syphilis).

### Sample collection

Blood specimens were collected via venipuncture into acid-citrate-dextrose tubes and processed using centrifugation at the on-site study laboratory within 6 hr of being drawn. The separated plasma was frozen at -20°C and the blood cells at -80°C. Vaginal lavage samples and pap smears were collected from all patients. Pap smears were examined and classified according to the pap classification protocol; pap I (normal), pap II (inflammation), pap III (dysplasia), pap IV (carcinoma in situ), and Pap V (carcinoma). For the purposes of the current study, only samples from patients with normal pap smears were analyzed for HPV. Vaginal lavage specimens were stored at -20°C. All specimens were then shipped to the Nebraska Center for Virology at the University of Nebraska-Lincoln (UNL) for sera and lavage testing.

### DNA isolation

Seventy cervico-vaginal lavage samples stored at -80°C were thawed, centrifuged and supernatant discarded. DNA was extracted using the Qiagen Tissue extraction kit (Dneasy). The DNA concentration was determined by UV spectrophotometer at 260 nm.

### PCR and gel electrophoresis

The quality of DNA isolation of was tested by β-actin amplification in all the 70 samples. HPV detection was carried out in all 70 samples using two sets of primers: GP5+(5'-TTTGTTACTGTGGTAGATACTAC-3'), GP6+ (5'-GAAAAATAAACTGTAAATCATATTC-3') and CPI (5'-TTATCWTATGCCCAYTGTACCAT-3'), CPII (5'-ATGTTAATWSAGCCWCCAAAATT-3') [25]. The conditions used for both primer sets were as follows: 1.5 mM MgCl_2_, 100 μM deoxynucleotide triphosphate (dNTP), 100 pmoles of each primer and 2.5 U of *Taq *polymerase (Invitrogen). A total reaction of 50 μl containing 1 μl template DNA was amplified according to the following PCR program of 94°C for 5 min followed by 40 cycles (95°C for 30s, 44°C for 1 min, 72°C for 90s) and 72°C for 10 min[25]. HPV16 plasmid DNA (pEF399) was used as positive control and water was used as a negative control. Discordant and negative samples were repeated using 1 μl, or 5 μl of template DNA, as necessary. Twenty-five μl of the PCR reaction was applied to 2% agarose gel and electrophoresed at 100 V for 105 min and stained in ethidium bromide. Positive bands were excised from the gel and the QIAquick Gel Extraction Kit protocol was used to purify the DNA (QIAGEN Inc. CA, USA). The pGEM-T vector system (Promega Corporation. WI, USA) was used to clone 3 μl of PCR product in a 10 μl reaction using the manufacturer's protocol. The reactions were incubated at 4°C overnight for maximum number of transformants. LB Media/ampicillin/isopropyl-beta-D-thiogalactopyranoside (IPTG)/5-bromo-4-chloro-3-indolyl-beta-D-galactopyranoside (X-GAL) plates were prepared (10 μl (200 mg/ml) of IPTG, 40 μl (20 mg/ml) of X-GAL). Locally prepared *E. coli *competent cells (75 μl/plate) were used and were incubated at 37°C overnight. At least three white colonies were selected from each plate and cultured in 5 ml LB/ampicillin (100 μg/ml) broth overnight at 37°C. Purification of plasmid DNA was carried out using QIAprep Spin Miniprep Kit (QIAGEN Inc. CA, USA), according to the protocol. Plasmid DNA was stored at 4°C for future use.

### HPV genotype identification

The sequence data from both CPI/CPII and GP5+/GP6+ PCR products was used to determine the HPV genotypes by conducting a BLAST search analysis against the NCBI database of viral DNAs. The genotype for each sample was determined with at least 100 nucleotides of DNA sequence.

### HIV tests

Plasma was tested in the study laboratory designated for HIV diagnosis blinded from HPV testing and diagnosis. HIV-1 serological status was established using two rapid assays, Capillus (Trinity Biotech, Bray Co., Wicklow, Ireland) and Determine (Abbott Laboratories, Abbott Park, IL), following manufacturers' suggested procedures. Plasma that tested positive by Capillus assay was confirmed by a Determine assay and vice versa. We were blinded as to the HIV status of all of the patients until after all HPV tests were completed.

### Statistical analysis

Classification of HPV types into high and low-risk categories were assessed according to previous descriptions. High-risk HPV types included: 16, 18, 26, 31, 33, 35, 39, 45, 51, 52, 56, 58, 59, 68, 73, 82, and 83 [6,9,26]. The primary goals of statistical analysis were to record the dispersal of HR and LR HPVs, characterize prevalence of HPV genotypes associated with this Zambian group, infer the presence, or lack of a relationship between HIV and HPV infections, and assess effects of HIV infection on distribution of HPV genotypes and perhaps the rate of dual HPV infections.

*Presence of any HPV *(Table 1) for a given socio-demographic characteristic did not account for multiple infections. Multiple infections were treated as presence of HPV and participants were not counted multiply for presence of more than one HPV. *HR and LR HPV prevalence *(Table 1) for soci-demographic characteristics were calculated as a function of HR or LR infections for total number of participants. Multiply infected participants thus accounted for more than one HPV infection but only one participant. Chi-Squared derived *P *values were also given in Table 1 to represent the significance of the statistical variation. To ensure that statistically significant differences could be detected with the sample size, a power analysis was performed using the G*Power program for Macintosh.

**Table 1:** Socio-demographics and diseases in relation to HPV prevalence.

		**Prevalence of HPV, %**					
		
Characteristic	**Number (%)**	**Any HPV**	***P *value**	**HR HPV**	***P *value**	**LR HPV**	***P *Value**
Total	70(100)	65.4		50.0		22.9	
**HIV Status**							
HIV+	30(45)	80.0	0.05	70.0	0.05	26.7	0.2
HIV-	40(55)	55.0		35.0		20.0	
**Age in years**							
15–22	30(42)	45.0	0.001	33.3	0.001	13.3	0.025
23–30	27(45)	64.0		59.3		33.3	
31–38	8(12)	78.0		75.0		12.5	
**Tribal Identity**							
Bemba	25(37.9)	60.0	0.001	48.0	0.001	20.0	0.001
Kaonde	2(3.0)	50.0		50.0		0.0	
Lozi	4(6.1)	100.0		75.0		50.0	
Luvale	1(1.5)	0.0		0.0		0.0	
Nyanja	24(36.4)	45.8		37.5		16.7	
Tonga	10(15.1)	80.0		70.0		30.0	
**Marital Status**							
Married	57(90.5)	63.2	0.001	50.9	0.001	24.6	1.0
Single	4(6.3)	50.0		50.0		0.0	
Live-in partner	2(3.2)	50.0		50.0		0.0	
**Education Status**							
1–7 years	35(54.7)	68.6	0.2	57.1	0.2	22.9	0.001
8–12 years	25(39.1)	56.0		44.0		20.0	
>12 years	4(6.2)	25.0		25.0		25.0	
**Age of Sexual Debut**							
≤ 15	19(28.8)	57.9	1.0	42.1	1.0	21.1	1
16–17	18(27.3)	61.1		44.4		27.8	
18–19	13(19.7)	76.9		69.2		23.1	
≥ 20	16(24.2)	43.8		43.8		12.5	
**Frequency Of Sex**							
Daily	1(1.5)	100.0	0.001	100.0	0.001	0.0	0.001
Once per week	6(9.2)	83.3		66.7		33.3	
Few per week	53(81.6)	69.8		58.5		24.5	
Few per month	2(3.1)	50.0		0.0		50.0	
Few per year	3(4.6)	33.3		33.3		0.0	
**No Contraceptive ever used**							
Yes	34(51.5)	58.8	1.0	47.1	1.0	17.6	1
No	32(48.5)	59.4		50.0		25.0	
**Use of Condom with Stable Partner**							
Always	1(1.5)	100.0	0.001	0.0	0.001	100.0	0.001
Frequently	4(6.1)	50.0		25.0		75.0	
Occasionally	14(21.2)	57.1		50.0		21.4	
Never	47(71.2)	59.6		51.1		14.9	
**Presence of Traditional Scarification**							
Present	17(25.8)	70.6	0.001	64.7	0.001	17.6	0.1
Absent	49(74.2)	53.1		40.8		22.4	
**Hematinics**							
Yes	42(62.7)	52.4	0.025	42.9	0.025	19.0	0.1
No	25(37.3)	68.0		56.0		24.0	
**History of Antibiotic Use**							
Yes	36(55.4)	61.1	1.0	50.0	1.0	22.2	1
No	29(48.5)	55.2		70.0		20.7	

The total number of various characteristics are indicated in response to the study questions (Numbers (%)). The prevalence of HPV (Any HPV) varies significantly across categories (*P *< 0.05, χ^2 ^test). The prevalence of low-risk HPV (LR HPV) varied significantly across categories (*P *< 0.05, χ^2 ^test). The prevalence of high-risk HPV (HR HPV) also varied significantly across categories (*P *< 0.05, χ^2 ^test).

## Results

### Demographic characteristics

Seventy patients from ages 15 to 38 years old, with normal cytology and verified HPV test results, were utilized for analysis. Among the 70 patients, 8 tested positive for the presence of multiple HPVs. HPV data was analyzed in the following tables and figures as infectious events, resulting in patients multiply infected being counted more than once.

To ensure that differences between conditions could be distinguished, a power analysis was performed using a sample size of 70 with an effect size of 0.5 and alpha set to 0.05. The resulting power calculated was 0.99. Given a sample size of 70, a Chi-Squared test at an alpha of 0.5 will have the power to detect a difference of at least 3.8 in the samples.

Socio-demographic characteristics of the study group are represented in Table 1. Median age of study participants was 22 years of age with a standard deviation of 5.2. Study participants median age of first sexual intercourse (sexual debut) was 17 years of age (standard deviation of 2.3).

Zambia has three predominant tribal identities, Bemba, Nyanga, and Tonga. These majority identities were represented in the study cohort. The majority Bemba tribe was represented in the study by 37.9% of the participants, whereas, Nyanga and Tonga tribes were represented in the study group at 36.4% and 15.1%. Other minority tribes, Kaonde, Lozi, and Luvale, were represented in the study at significantly lower numbers. An overwhelming majority of study participants, over 90%, reported themselves as married (formal or common-law marriage). Approximately 35% attended or completed only primary education (1–7 years), whereas 25% had secondary education (8–12 years) and 6.2% had received some form of tertiary education.

Behavioral risk factor assessment included frequency of intercourse, and use of contraception. Approximately 81.6% reported having sexual intercourse at least a few times per week, while 3.1% reported having intercourse a few times per month. Use of contraception was evaluated on the basis of a participant utilizing some form of contraception at least once. About half (51.5 %) of the participants had never used any form of contraception and half (48.5%) had utilized contraception. The majority of participants never used condoms (71.2%) with a stable partner and a substantial fraction (27.3%) sporadically utilized condoms.

### Socio-demographic characteristics in reference to HPV prevalence

The overall prevalence of HPV among study participants was 65.4% and 45% for HIV. HPV prevalence in HIV positive participants was 80%, and 55.0% in HIV negative participants (Table 1). The prevalence of HPV HR and LR and infections was calculated in relation to the population characteristics. Multiple infections were observed in 11.4% of the study participants, 7.1% of those observed in HIV positive patients and 4.3% in HIV negative patients. HR and LR HPV infection prevalence for all participants was 50% and 22.9%, respectively (Table 1). HIV positive patients had a frequency of HR HPV infection at 70.0% and LR HPV infection frequency of 26.7%, whereas HIV negative patients were found to have an infection frequency of 35% for HR and 20% for LR HPVs.

### HPV genotype distribution

The incidence of both HR and LR genotypes was determined in both HIV positive and negative patient samples to determine if HIV status influences the distribution of HPV genotypes (Table 2). We discovered an obvious increase in the frequency of HR HPVs, specifically HPV18, in HIV positive versus HIV negative patients. However, We found that the incidence of low-risk HPVs was unaffected by HIV status.

**Table 2:** The frequency of HPV genotypes in HIV positive and negative patients.

**HPV Genotype**	**HIV+ Group**		**HIV- Group**		**Total**	
	**HPV Frequency**	**Percent**	**HPV Frequency**	**Percent**	**HPV Frequency**	**Percent**

**High Risk**						
**16**	4	13.8	7	31.8	11	21.6
**18**	10	34.5	1	4.5	11	21.6
**58**	1	3.4	1	4.5	2	3.9
**35**	1	3.4	1	4.5	2	3.9
**45**	1	3.4	0	0	1	2.0
**51**	1	3.4	0	0	1	2.0
**50**	1	3.4	1	4.5	2	3.9
**83**	1	3.4	0	0	1	2.0
**67**	1	3.4	0	0	1	2.0
**33**	0	0	2	9.1	2	3.9
**8**	0	0	1	4.5	1	2.0

**Total**	**21**		**14**		**35**	

**Low Risk**						
**6**	2	6.9	2	9.1	4	7.8
**3**	1	3.4	1	4.5	2	3.9
**54**	1	3.4	1	4.5	2	3.9
**43**	2	6.9	1	4.5	3	5.9
**91**	1	3.4	0	0	1	2.0
**45**	1	3.4	0	0	1	2.0
**62**	0	0	1	4.5	1	2.0
**74**	0	0	1	4.5	1	2.0
**81**	0	0	1	4.5	1	2.0

**Total**	**8**		**8**		**16**	

**HPV Negative**	**6**		**18**		**24**	
**HPV Positive**	**29**		**22**		**54**	
**Number of Samples**	35		40		75	

The incidence of each HPV genotype was determined and the percent total representation of each was calculated. Genotypes were recorded on a per incident basis since several patients had multiple HPVs. The data is represented graphically in figure 2.

**Figure 1:**
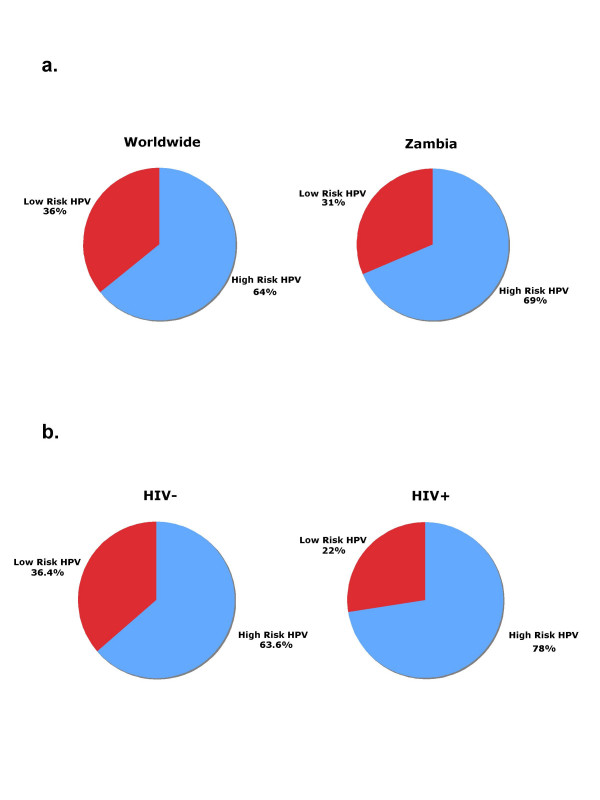
**a**. HPV infections are graphed by risk type comparing worldwide and Zambian HPV distributions. The worldwide HPV distribution data was adapted from Clifford *et al*. (2005) and Peyton *etal*. (2001) [4] [5] [See Additional file 1]. High-risk (HR; blue) and Low-risk (LR; red) HPV distributions among Zambian and worldwide populations. **1b. **HPV infections by risk type in relation to HIV status. High-risk (HR; blue) and Low-risk (LR; red) HPV distributions are graphed as a function of HIV status for Zambian study participants. The distribution of HR and LR HPVs is displayed as the percent of HPV infections in participants. Positive HIV status is associated with a higher prevalence of HR HPV infections.

Figure 1a shows the distribution of HPV risk types in reference to worldwide data and Zambia. Worldwide HPV data was adapted from G.M. Clifford and C. M. Wheeler [4,5]. The two studies were utilized to assess worldwide distribution of HPV types. HPV type prevalence data from the US study was normalized to regional type prevalence data collected in the Clifford et al. worldwide HPV study [See Additional file 1]. Normalization of worldwide data produced HR/LR distributions to be 64% and 36% respectively. Similarly, the observed Zambian distribution for HR HPVs was 68.6% and 31.4% for LR HPVs. The correlation of HIV status with HPV risk type distribution among the Zambian cohort is represented in Figure 1b. HIV negative participants were found to have risk-type distributions similar to the larger worldwide population. The HPV distribution for HIV negative participants was 63.6% HR to 36.4% LR. HIV positive participants displayed a significantly higher distribution of HR HPVs than that found in the HIV negative group or in the worldwide data. The HR HPV distribution was shown to be 72.4% and LR HPV was present in 27.6% of HPV infections for the HIV positive group of Zambian patients.

**Figure 2:**
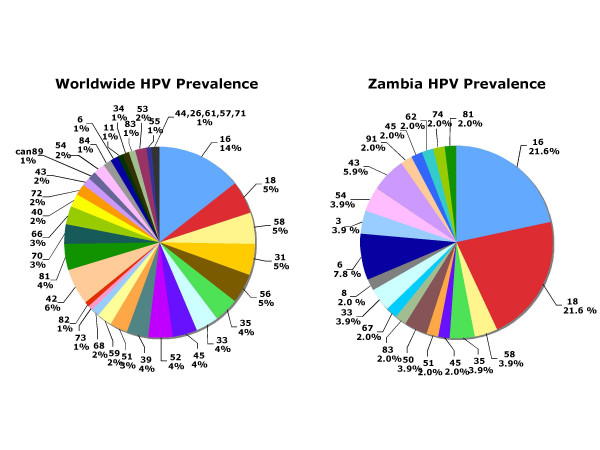
The frequency of HPV genotypes in Zambia as compared to the worldwide HPV distribution. The worldwide HPV data was adapted from Clifford *et al*. (2005) and Peyton *et al*. (2001) [4] [5]. Both HPV16 and HPV18 were present at 21.6% compared to 14% and 5%, for HPV16 and HPV18, respectively.

Genotype frequencies from the worldwide HPV approximation and Zambia are shown in Figure 2. Worldwide, HPV16 has been shown to the predominating HPV genotype with a 14% frequency. Other prominent HR genotypes worldwide include HPV18, 31, 56, and 58; all of which are present at a frequency of 5% worldwide. The Zambian cohort was found to have two predominating HR genotypes, HPV16 frequency 21.6% and HPV18 frequency 21.6%. HPV18 frequency was approximately four-fold higher in the Zambian cohort than that reported worldwide.

**Figure 3:**
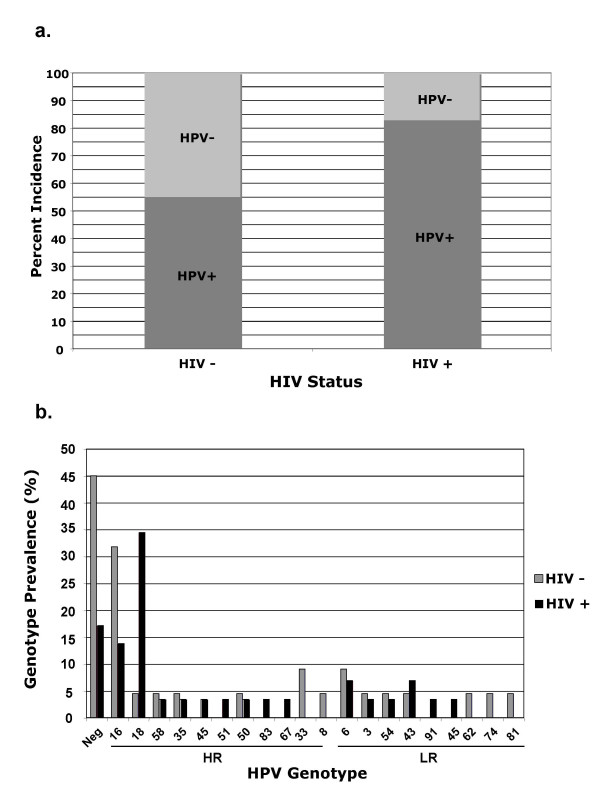
**a **The incidence of HPV was calculated as a function of HIV status. The data was normalized as percent incidence to assesses possible effects or associations of HIV status with HPV infection. **3b. **The prevalence of HPV genotypes present in patients was assessed for both HIV positive (black) and negative patients (gray). The graph displays HPV genotypes on the x-axis and the frequency of each genotype on the y-axis. HR indicates high-risk strains and LR, represents the low-risk strains.

Frequency of HPV genotypes was also assessed according to HIV status (Figure 3b). Genotype frequencies associated with HIV negative status was comparable to genotype frequencies observed worldwide, in that HPV16 was the predominating HPV genotype present. HIV positive status was associated with a nine-fold higher frequency of HPV and a moderate decline in HPV16 frequency compared to the HIV negative group. Age-related affects do not explain these differences since both HIV positive and negative patients had the same mean and median ages (22 years old). The nine-fold increase in HPV18 frequency among HIV positive participants accounted, almost solely, as the primary factor for the higher prevalence of HR HPV types in HIV positive participants. This difference was significant with a Chi-squared of 6.38, at a *p*-value of 0.025.

## Discussion

In this study we investigated the distribution of HPVs in a population of Zambians who were patients of the UTH in Lusaka. The average rate of HIV infection in Zambia is approximately 25.4% in urban areas and 11.5% in rural areas [27]. However, in our study group, 45% of patients were HIV positive. It is reasonable that this high rate of HIV infection indicates that this population is at a higher relative risk for sexually transmitted disease. Patients who are admitted to UTH are aware that they are having health problems and often this is indirectly related to their HIV status.

There appeared to be a number of socio-demographic factors that were predictive of HPV status (Table 1). Older patients, between the age of 23–30 or 31–38, were at least two-fold more likely to be infected with an HR HPV than patients who were 15–22 years old. Similarly, patients between the age of 23–30 were two-fold more likely to be infected with an LR HPV than the 15–22 year old group. Most of our patients (90.5%) were married.

There appeared to be a moderately greater likelihood that a given patient was HR HPV infected if they had a lower education level (1–7 years) versus 8–12 years. The age of sexual debut was reliably predictive of HR HPV infection; patients who reported their first sexual experience between 18–19 years of age were most likely to be infected with an HR HPV. However, age of sexual debut appeared not to be significantly predictive of LR HPV infection.

We compared the ratio of HR to LR HPVs in Zambia to the worldwide distribution [4] (Figure 1a). We found that the worldwide distribution of HR (64%) and LR HPVs (36%) was very similar to that in Zambia; HR (69%) and LR HPVs (31%) [5]. This suggests that the Zambia population is not unusual with reference to the worldwide distribution. However, There was a significant effect of HIV on the percentage of HR HPV infection detected (Figure 1b). In HIV positive individuals the HR to LR ratio was 78% to 22% versus 63.6% to 36.4% in HIV negative individuals. This effect has been observed by other studies [28] and it is clear that the effect of HIV on the frequency of HPV infection is likely to be related to HIV-dependent immune suppression [17,29,30].

We identified HPV DNAs in vaginal lavage samples by amplification using GP5+/GP6+ and CPI/CPII primer sets. We discovered that HPV16 and HPV18 each were present in 21.6% of Zambian samples (Figure 2). The aggregate worldwide rates of HPV16 and 18 are 14% and 5% respectively. Thus, HPV18 is present in this Zambian population at about four-fold higher than the average world rate [4]. The approximate rates of HPV16 and HPV18 in the US are 7.5% and 2.3%, respectively [5]. In Zambian patients, this corresponds to about a three-fold and nine-fold greater incidence of a HPV16 and HPV18, respectively. This finding, potentially, explains the high rates of cervical cancer in Zambia and other sub-Saharan countries [23,31-34]. As other laboratories have reported, we observed a significant increase in HPV incidence as a function of positive HIV status [13,32,33,35,36]. Specifically, we observed that incidence of HPV was 55% in HIV negative patients whereas, the HPV incidence in HIV positive individuals was 80% (p = 0.05) (Figure 3a). This probably represents increased replication efficiency of viruses in patients with compromised immune systems.

Although, intuitively, it would seem reasonable that all HPVs should increase in frequency in HIV positive individuals, we observed a non-random increase in the incidence of certain HPV genotypes. We found a nine-fold increase in the incidence of HPV18 in HIV positive versus HIV negative patients (Figure 3b). Despite these differences in HPV distribution, all of the women in our study had normal pap smears, suggesting that HIV status alters HPV infection at levels of disease that are clinically unapparent. Other studies have witnessed this effect for HPV18 in the presence of HIV infection, though no studies have provided explanations. Studies in Zimbabwe by Baay and colleagues observed a three-fold increase in HPV18 incidence in HIV positive individuals [13,15], similar to our observations. The depletion of memory T cells by HIV could be responsible for the genotype specific differences due to loss of critical portions of the T cell receptor repertoire.

A recent study by Parham et al. [37] in Zambian patients at the UTH found a strong correlation between low CD4 counts (<200 cells/μl) and the odds of finding severe cytological abnormalities. In contrast to our studies, all of the patients in their study were HIV positive and the rates of high-grade lesions was also high. While in our study, we observed 69% of the patients infected with HR HPVs, the Parham study reported 85%, a result which is likely to be consistent with risk-level in HIV positive patients. The presence of cytological abnormalities in HIV positive patients is, not surprisingly, predictive of patients being positive for an HR HPV and vice versa [37].

The comprehensive study by Clifford et al. [4] compared HPV genotype distributions in Nigeria, India, Vietnam, Thailand, Korea, Colombia, Argentina, Chile, the Netherlands, Italy, and Spain. The patients in this study were not tested for HIV. However, the authors speculated that regional differences in the distribution of HPVs other than HPV16 could be partly explained endemic HIV, particularly in Nigeria and India. Out of 3230 patients with no cytological abnormalities, the prevalence for any HPV was 36.3% and 11.9% for multiple HPV types. The six most common high-risk HPV types were 16 (4.5%), 58 (3.6%), 18 (3.1%), 52 (2.8%), 31 (2.0%) and 33 (2.0%) [4]. In our Zambian study population, we discovered pronounced differences in HPV distribution from the study of Clifford et al. [4]. For example, the most common HPV types in our study were 16 (21.6%), 18 (21.6%), 6 (7.8%), 43 (5.9%), 58 (3.9%), 35 (3.9%), 50 (3.9%), 54 (3.9%), 33 (3.9%) and 3 (3.9%). Furthermore, HPV type 31 was not recovered in our patient group. Thus, we conclude that the distribution of HPVs in this population differs significantly from the worldwide and US averages [4,5] and that this is influenced by HIV status. The study by Clifford et al (2006) found that HIV-positive women with HSIL were significantly more likely to be infected with HPV types 11, 18, 33, 51, 52, 53, 58 and 61, and with multiple HPV types [38]. These results are consistent with our observations. Our study made use of standard PCR methods, similar to those of Peyton et al. (2000) and Clifford et al. (2005) [4,5]. In our patient group, we had an HIV positive rate of 45%. The most dramatic difference attributable to HIV that we noted was a statistically significant increase in HPV18 in HIV positive patients.

## Conclusion

In conclusion, our studies suggest a very active interaction between HIV and HPV that is likely to de-repress the replication of HR HPVs (HPV18; figure 3b). The high rates of HPV16 and 18 in Zambia are several-fold higher than those found in the US and likely to be, at least partly, due to the prevalence of HIV infection and the immunosuppressive effects of HIV. This also raises the question of whether there is increased rate of transmission of particular HR HPVs in couples in which one or both of the partners is HIV positive.

## Competing interests

The author(s) declare that they have no competing interests.

## Authors' contributions

CN performed the majority of the experiments. JJL performed the data analysis and helped prepare the manuscript. PJR and LH performed genotyping experiments. CW provided patient samples, logistical support, organized collaborators in Zambia. PCA provided experimental, logistical and financial support and helped to prepare the manuscript. The authors have read and approved the final manuscript.

## Pre-publication history

The pre-publication history for this paper can be accessed here:



## Supplementary Material

Additional file 1 Appendix – Detailed explanation of the methods of calculations of worldwide HPV genotype averages in Ng'andwe et al. A detailed explanation of how a worldwide average for HPV genotypes was calculated. Click here for file
