# Supplementary material for: The distribution of sexually-transmitted Human Papillomaviruses in HIV positive and negative patients in Zambia, Africa
Source: BMC Infect Dis. 2007 Jul 16;7:77. doi: 10.1186/1471-2334-7-77 (PMC1949816; doi:10.1186/1471-2334-7-77)
Supplement: Additional file 1 — Appendix – Detailed explanation of the methods of calculations of worldwide HPV genotype averages in Ng'andwe et al. A detailed explanation of how a worldwide average for HPV genotypes was calculated. [file 1471-2334-7-77-S1.doc]

**Detailed explanation of the methods of calculations of worldwide HPV genotype averages in Ng’andwe et al.**

Worldwide data graphs were adapted (with permission) from data collected by Clifford et al. and Peyton et al. Peyton data was normalized to fit the Clifford study in a region based method.

Raw data obtained from Clifford’s *Worldwide distribution of human papillomavirus types in cytologically normal women in the International Agency for Research on Cancer HPV prevalence surveys: a pooled analysis,* Table 2 from Clifford et al. depicts raw data for type specific HPV prevalence observed (1).

In Clifford et al. Table 2, they observed 2003 HPV infection and 25 of those were unable to type. These un-typed infections were not included in our analysis of the data. Table 1 of Clifford (1) represents HPV prevalence by study age and study area (country and region). The area/region data represented in Clifford Table 1 was utilized to establish the weight that should be given in normalizing Peyton et al. data to the Clifford et al. study (1).

The *Number with any HPV* data represented in Clifford Table 1 was used to calculate the mean number of HPV positive patients by region/area: 357.25.

Peyton et al. HPV type percent prevalence data (Peyton Table 1) was then normalized to the Clifford study by weighting Peyton et al. percent prevalence to the mean number of infected individual observed per area/region of Clifford et al (2).

For example, the following calculation was performed to normalize Peyton’s HPV 16 percent prevalence to the Clifford data. For example:

.075 x 357.25 = 26.79 infections

This normalized number of HPV 16 infections was then summed with the Clifford number of HPV 16 infections represented in Clifford Table 2: 282

26.79 + 282 =308.79

This “Peyton/Clifford Sum” was then used to calculate genotype frequencies as a function of “Peyton/Clifford Sum” of all types present.

308.79 ÷ 2121.85 = 14.55%

This procedure was followed for every HPV type present.

**References**

1. **Clifford, G. M., J. S. Smith, M. Plummer, N. Munoz, and S. Franceschi.** 2003. Human papillomavirus types in invasive cervical cancer worldwide: a meta-analysis. Br J Cancer **88:**63-73.

2. **Peyton, C. L., P. E. Gravitt, W. C. Hunt, R. S. Hundley, M. Zhao, R. J. Apple, and C. M. Wheeler.** 2001. Determinants of genital human papillomavirus detection in a US population. J Infect Dis **183:**1554-64.
